# Climate Change or Land Use Dynamics: Do We Know What Climate Change Indicators Indicate?

**DOI:** 10.1371/journal.pone.0018581

**Published:** 2011-04-21

**Authors:** Miguel Clavero, Daniel Villero, Lluís Brotons

**Affiliations:** 1 Grup d'Ecologia del Paisatge, Àrea de Biodiversitat, Centre Tecnològic Forestal de Catalunya, Solsona, Catalonia, Spain; 2 Departament de Ciències Ambientals, Universitat de Girona, Girona, Catalonia, Spain; 3 European Bird Census Council and Institut Català d'Ornitologia, Museu de Ciències Naturals, Zoologia, Barcelona, Catalonia, Spain; University of Durham, United Kingdom

## Abstract

Different components of global change can have interacting effects on biodiversity and this may influence our ability to detect the specific consequences of climate change through biodiversity indicators. Here, we analyze whether climate change indicators can be affected by land use dynamics that are not directly determined by climate change. To this aim, we analyzed three community-level indicators of climate change impacts that are based on the optimal thermal environment and average latitude of the distribution of bird species present at local communities. We used multiple regression models to relate the variation in climate change indicators to: i) environmental temperature; and ii) three landscape gradients reflecting important current land use change processes (land abandonment, fire impacts and urbanization), all of them having forest areas at their positive extremes. We found that, with few exceptions, landscape gradients determined the figures of climate change indicators as strongly as temperature. Bird communities in forest habitats had colder-dwelling bird species with more northern distributions than farmland, burnt or urban areas. Our results show that land use changes can reverse, hide or exacerbate our perception of climate change impacts when measured through community-level climate change indicators. We stress the need of an explicit incorporation of the interactions between climate change and land use dynamics to understand what are current climate change indicators indicating and be able to isolate real climate change impacts.

## Introduction

Climate change is altering biological processes and having important impacts on biodiversity at multiple scales [1], [2]. However, the responses of species and biological communities to climate change can also be influenced by the additive or synergistic effects of other components of global change, such as land use changes or biological invasions [3]. In fact, the magnitude of the impacts of each one of the different components of global change, and therefore their interactions, is subjected to variation among systems and biomes [4], [5], [6].

Both scientists and policy practitioners are seeking valid compound indicators to track the complex impacts of climate change on biodiversity [7], [8]. An appealing approach has been the integration of information across species, including species' distributions or abundances and their thermal optima, to summarize responses to climate change at the local community level. In this line, recent works have proposed indicators to describe community changes associated with climate warming, reflecting a generalized trend towards a higher representativeness of high-temperature dwelling species [9]. However, climate change indicators could also be influenced by other co-occurring global change processes, such as land use changes or modified disturbance regimes. If mean climatic envelope of species within communities varied as a function of land uses, climate change indicator figures would be affected by factors other than climate change in a dynamic global change scenario. In spite of this, the potential critical role of the interactions among components of global change on our perception of climate change impacts through community indicators has not yet been explicitly analyzed.

There have been multiple insights on the habitat associations of the characteristics of the climatic niche of bird species in the Mediterranean region, where land use changes are thought to be one of the main drivers of biodiversity loss [4]. Results of these works report that forest communities have species with more northern distributions ranges [10], [11] and are dominated by cold-dewelling species [12]. Thus, it seems plausible that climate change indicators based on the average characteristics of the climate niche within communities would be related to habitat characteristics. Given the dynamic nature of landscapes due to land use changes, to what extent can we conclude that variation in community based climate change indicators is entirely induced by climate change?

In this work, we use data on the composition of bird communities to analyze the variation of different climate change indicators along land use gradients while controlling for the thermal environment. Our primary aim is to describe the influence of the main current landscape dynamics on climate change community indicators. This information should serve as a basis to incorporate different components of global change in the design of indicators of trends in biological diversity.

## Methods

### Bird data and community indicators

This study was carried out in Catalonia, a Mediterranean-climate area located in NE Iberian Peninsula. We obtained data on the occurrence of 127 diurnal, terrestrial bird species from the Catalan Breeding Birds Atlas [13], which reports information on breeding bird distribution in Catalonia between 1999 and 2002 based on intensive surveys of 3077 1×1 km grid cells.

We estimated the optimal thermal environment of each species through the species temperature index (STI). STI values report the average mean temperature (in °C) experienced by each species during the breeding season (March to August) across its distribution range [9]. Although STI values produce a reliable ordination of the thermal environment of species in a given area, they are scale-dependent and site-specific (e.g. they would be larger in southern areas). Thus, we calculated STIs at two different scales: regional (using data from Catalonia, henceforth STIcat) and continental (using data from Europe, henceforth STIeur). STIcat was based 1×1 km cells data from the Catalan Breeding Birds Atlas [13], while STIeur used 50×50 km cells data from the EBCC Atlas of European Breeding Birds [14]. Information on temperatures was derived from the Worldclim database (http://www.worldclim.org). Furthermore, since the distribution range of many of the species included in the analyses extents well beyond the European borders, we used average latitude (AL) as an additional indirect descriptor of climate niche of bird species. AL values were compiled by Prodon [10], from which we used AL that were calculated using only the Old World distribution of each species.

We averaged STI figures for species occurring in each 1×1 km cell surveyed in Catalonia to obtain community temperature indexes (CTIs). CTIs report the average breeding-season temperature optimum of species in a given local community. The CTI has been proposed as a climate change indicator to quantify trends in the patterns of community composition in response to global warming, both for birds [9] and butterflies [15]. We calculated both regional and continental CTIs (CTIcat and CTIeur, respectively). We also averaged AL of species occurring in 1×1 km cells, to obtain a community average latitude (CAL). Larger CAL values would thus indicate communities dominated by species with more northern distributions.

### Landscape gradients

At each 1×1 km cell, we defined landscape characteristics through four variables: the percentage cover of forests, agricultural and urban uses (in %, from the 1997 Catalan land use map) and the percentage of each cell burnt by wildfires in the period 1986–1999, calculated from fire perimeters [16]. From these variables we created three landscape gradients corresponding to land use changes which have had stronger impacts in Mediterranean landscapes in last decades: i) farmland to forest (land abandonment); ii) wildfire to forest (fire impact); and iii) urban to forest (urbanization) [17], [18], [19]. To construct each of those gradients, we selected cells in which the sum of the two variables involved in the gradient was ≥75%. For the urban to forest gradient we further selected cells in which urban uses cover was ≥25%. We derived final gradients by subtracting the percentage cover of agricultural, burnt or urban areas to that of forests, so the gradients varied from −100 (completely agricultural, burnt or urban cells) to 100 (completely forested cells) [12]. The land abandonment and fire gradients included cells with elevations up to 1000 msl, while the altitude limits of the urbanization gradient was 700 msl. We set elevation limits attending to the representativeness of both gradients' extremes, in order to avoid confounding effects of altitude and landscape gradients (see supporting information, Figure S1). For example, since wildfires are rare at altitudes higher than 1000 m, if the landscape gradient included forests up to 2000 m, there would be a strong relationship between altitude and the fire gradient.

### Data analyses

In a first step, we analyzed the relationships among the different indicators, using simple correlation analyses. We run correlation analyses both at the species (STIcat, STIeur and AL) and the community (CTIcat, CTIeur and CAL) levels, using, respectively, species and 1×1 km cell as samples.

Then, we analyzed the variation of the different community indicators (CTIcat, CTIeur, CAL) along landscape and temperature gradients, using linear regression models. For each indicator and landscape gradient, we first run two simple regression models alternatively using landscape gradient or breeding-season temperature site values as predictors. In a second step we run a multiple regression model including both predictors. Simple and multiple regression models were compared by differences in the coefficient of determination (*R* ^2^) and through the Akaike information criteria (AIC). We considered a specific regression model (whether simple or multiple) as the most adequate one if its AIC was at least 7 points lower than that of the other models run for a given community indicator and landscape gradient. We assessed the strength of the associations between the different indicators and each model term (temperature and landscape gradients) through the examination partial Eta squared (η_p_^2^) [effect sum of squares (SS)/(effect SS + error SS)] [20]. This statistic is a measure of the size of the effects of model terms that is independent of the degrees of freedom used in the analyses.

We estimated the predicted change in the values of community indicators if a landscape changed from one extreme of a landscape gradient to the other (e.g. a forest becomes a farmland area, is burnt by a wildfire or is urbanized). To this aim we selected those 1×1 km cell placed at gradient extremes and classified them as: i) farmland, wildfire or urban (when gradient values were smaller than −75); or ii) forest (gradient values larger than 75). We used an analysis of covariance (ANCOVA) approach to test and quantify the influence of habitat (factor) on climate change indicators while controlling by average temperature during the breeding season (covariate). We first performed homogeneity of slopes analyses, and whenever the factor × covariate interaction was not significant (significance level set at *P*<0.01 due to large sample sizes), it was deleted from the model. The variation in climate change indicators values between the extremes of landscape gradients was assessed through their estimated marginal means at a given habitat, calculated at covariates' means (i.e. average thermal environments). Finally, we used regression equations from Catalonia, based on 2824 1×1 km cells with elevations up to 2000 m, or those previously published by others [9] to find spatial and temporal variations generating changes in climate change indicators equivalent to those induced by landscape transformation. Regression coefficients employed in those analyses are reproduced in Table S1.

## Results

**Figure 1:**
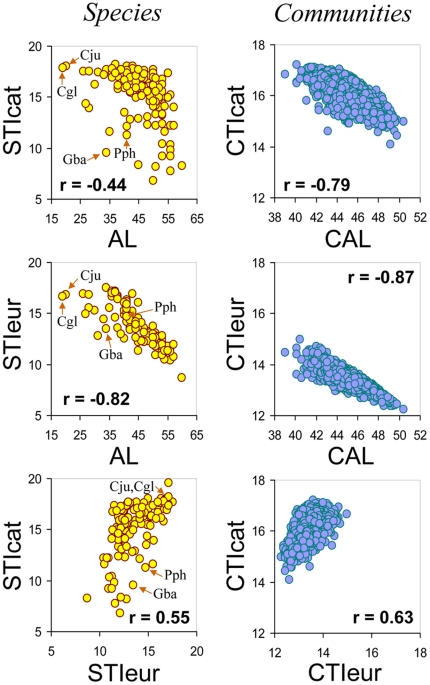
Relationships among climate change indicators at species and community levels. STIcat: species temperature index for Catalonia; STIeur: species temperature index for Europe; AL: average latitude of species' ranges; CTIcat: community temperature index for Catalonia; CTIeur: community temperature index for Europe; CAL: community average latitude of species' ranges. Correlation coefficients (Pearson's r) are given for each relationship. The positions of Zitting cisticola (*Cisticola juncidis*, Cju), great spotted cuckoo (*Clamator glandarius*, Cgl), bearded vulture (*Gypaetus barbatus*, Gba) and chough (*Phyrrocorax phyrrocorax*, Pph), which are commented in the text, are marked in the species graphics.

The different indicators were highly correlated, both at the species and at the community levels (Figure 1). However, STI values, whether regional or continental, tended to underestimate the optimal thermal environment of species with distribution ranges that extended southwards from European borders (e.g. zitting cisticola, *Cisticola juncidis*, or great spotted cuckoo, *Clamator glandarius*). Moreover, the regional STI (STIcat) of montane bird species was often lower than that expected from the average latitude of their distribution. This is the case of the bearded vulture, *Gypaetus barbatus*, or the chough, *Phyrrocorax phyrrocorax*, which occupy cold, mountainous environments in Catalonia while having on average quite southern ranges (average latitude 34° and 41° N, respectively). In spite of these differences at the species level, when information of species occurrences was pooled to create different community indicators, these were more strongly interrelated than species original data (Figure 1).

**Figure 2:**
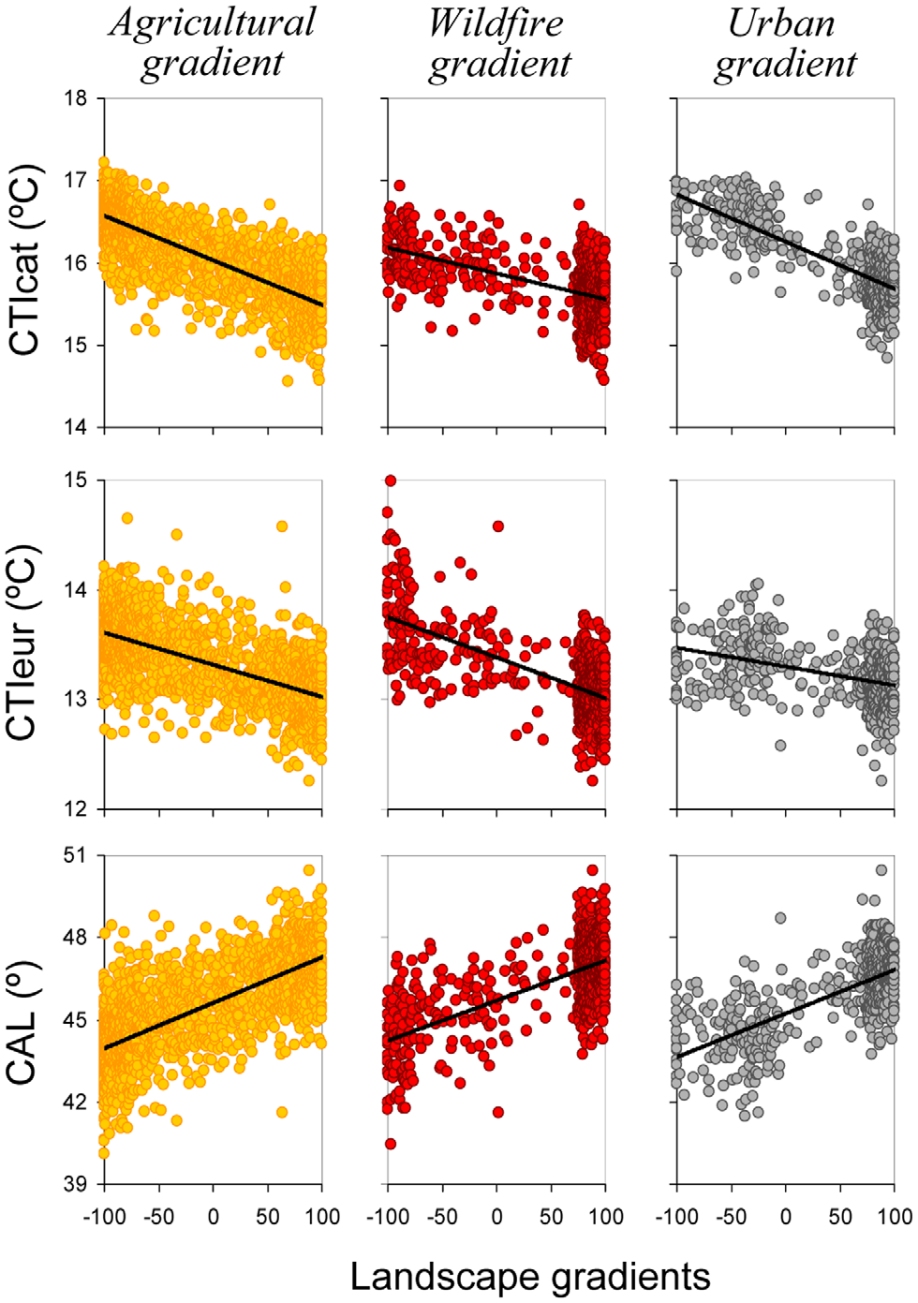
Linear relationships between landscape gradients and community-level climate change indicators. Indicators' codes as in Figure 1.

In eight out of nine groups of analyses (three indicators × three landscape gradients) the multiple regression model worked better than the simple regression ones (Table 1), differences in AIC with the second-ranking model being larger than 65 (range 65.4–743.8) in all eight cases. This implies that landscape gradients have a significant influence on the values of the climate change indicators once the effects of temperature have been taken into account. Moreover, the effects of landscape gradients in the multiple regression models (as measured by the η_p_^2^) were of the same magnitude, when not clearly larger, than those of temperature (Table 2). The sole exception to this general pattern is the variation of CTIeur along the urban gradient, which was better explained by temperature alone, although the difference in AIC with respect to the multiple regression model was only 2.8. Bird communities at the forest end of the three landscape gradients consistently tended to have colder-dwelling bird species as well as species with more northern ranges (Figure 2).

**Table 1:** Influence of thermal environment and landscape gradients on climate change indicators.

			CTIcat		CTIeur		CAL	
	Predictors	df	dir	*R* ^2^	dir	*R* ^2^	dir	*R* ^2^
Agricultural gradientN = 1431	Temperature	1	+	0.65	+	0.36	-	0.47
	Gradient	1	-	0.59	-	0.36	+	0.49
	Both (T, G)	2	**+, -**	**0.80**	**+, -**	**0.46**	**-, +**	**0.62**
Wildfire gradientN = 551	Temperature	1	+	0.47	+	0.21	-	0.24
	Gradient	1	-	0.30	-	0.43	+	0.42
	Both (T, G)	2	**+, -**	**0.64**	**+, -**	**0.54**	**-, +**	**0.55**
Urban gradientN = 439	Temperature	1	+	0.51	**+**	**0.23**	-	0.39
	Gradient	1	-	0.62	-	0.16	+	0.46
	Both (T, G)	2	**+, -**	**0.72**	+, -	0.25	**-, +**	**0.54**

Simple and multiple regression models analyzing the relationships between community-level climate change indicators and: i) average temperature; ii) landscape gradients; and iii) both independent variables. Coefficient of determination (*R* ^2^) values marked in bold are those of models having the strongest support after the Akaike information criterion (AIC). The direction (positive or negative) of relationships between independent variables and climate change indicators are also given.

**Table 2:** Slopes and effects' strength of the relationships between climate change indicators and temperature and landscape gradients.

		CTIcat		CTIeur		CAL	
	Predictors	*b*	η_p_^2^	*b*	η_p_^2^	*b*	η_p_^2^
Agricultural gradientN = 1431	Intercept	13.044		11.886		53.491	
	Temperature	0.179	0.50	0.086	0.15	-0.472	0.24
	Gradient	-0.003	0.41	-0.002	0.16	0.011	0.27
Wildfire gradientN = 551	Intercept	13.057		11.832		52.487	
	Temperature	0.175	0.48	0.096	0.19	-0.421	0.22
	Gradient	-0.002	0.32	-0.003	0.42	0.013	0.41
Urban gradientN = 439	Intercept	13.682		11.826		52.574	
	Temperature	0.148	0.27	0.085	0.11	-0.422	0.15
	Gradient	-0.004	0.43	-0.001	0.03	0.011	0.25

Regression coefficients corresponding to the multiple regression models shown in Table 1. Partial Eta squared values (η_p_^2^) are given as a measure of the strength of the effect of each model term.

**Figure 3:**
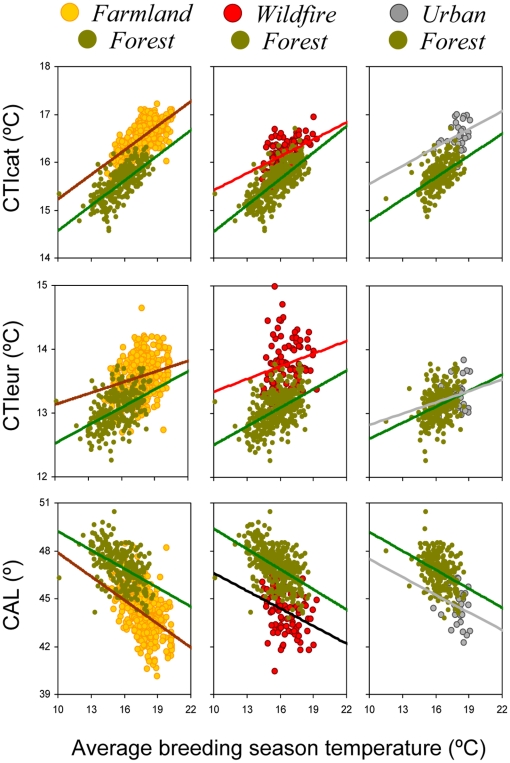
Linear relationships between average temperature during breeding season and community-level climate change indicators, shown separately for landscape gradient extremes. Indicators' codes as in Figure 1.

As shown by marginal means given in Table 3, if an abandoned farmland area became a forest climate change indicators would indicate a trend towards colder-dwelling, more northerly distributed bird communities. On the other hand, if a forest was burnt by a wildfire or urbanized, CTIcat and CTIeur would increase and CAL values would decrease (Figure 3). Using regression equations given in Table S1, we found that those changes in the values of climate change indicators would be equivalent to elevation changes of several hundred meters (up to more than 900 m) or to changes in temperature during breeding season averaging 2.9°C (Table 4). The average change in CTIeur between the extremes of landscape gradient extremes would also be equivalent to moving forward or backward up to more than one century of global warming effects on bird communities or to changes in latitude of several hundred kilometers, according to the patterns of variation recorded in France for the same community indicator (Table 4).

**Table 3:** Mean values of climate change indicators at the extremes of landscape gradients (i.e. farmland, burnt or urban areas and forest areas) at average temperature conditions.

	CTIcat (°C)		CTIeur (°C)		CAL (°)	
Habitat	mean	SE	mean	SE	mean	SE
Farmland	16.43	0.012	13.52	0.013	44.33	0.056
Forest	15.80	0.017	13.16	0.019	46.36	0.081
Wildfire	16.10*	0.031	13.71	0.030	44.48	0.118
Forest	15.62*	0.014	13.07	0.014	46.91	0.057
Urban	16.34	0.052	**13.17**	**0.050**	45.08	0.205
Forest	15.76	0.017	**13.15**	**0.016**	46.59	0.065

Values and their associated standard errors are marginal means derived from analyses of covariance (ANCOVAs) of data shown in Figure 3.

*denotes ANCOVAs in which the interaction term (habitat × temperature) was significant; otherwise the interaction was removed from the final ANCOVA model. Numbers in bold denote analyses in which the factor “habitat” did not have a significant effect on a climate change indicator.

**Table 4:** Spatial and temporal variations producing changes in climate change indicators equivalent to those observed between the extremes of landscape gradients.

			Equivalent to the following changesas measured by climate change community indicators			
Process	Habitat change under analysis	Indicator	Altitude Catalonia (m)	Temperature Catalonia (°C)	Years France	Latitude France (km)
Land abandonment	From farmland to forest	CTIcat	+386.6	-2.33		
		CTIeur	+522.1	-2.96	-81.8	+352.9
		CAL	+676.6	-3.87		
Fire impact	From forest to open areas/shrubland	CTIcat	-294.5	+1.77		
		CTIeur	-928.2	+5.25	+145.5	-627.5
		CAL	-809.9	+4.63		
Urbanization	From forest to urban areas	CTIcat	-355.9	+2.14		
		CTIeur	-29.0	+0.16	+4.5	-19.6
		CAL	-536.6	+3.07		

The variation of climate change indicators (from mean values given in Table 3) are here related to the main processes of land use changes occurring in Mediterranean landscapes in last decades: i) land abandonment; ii) fire impact; and iii) urbanization. Regression coefficients used to calculate spatial and temporal variations producing equivalent changes in climate change indicators are given in Table S1.

## Discussion

Our results clearly show that climate change indicators based on the composition of bird communities are dependent on land use characteristics. This is due to the variation in mean optimal thermal conditions of bird communities occupying different habitats. Thus, land use changes would likely produce increases or reductions in climate change indicator figures even in a theoretical, though unrealistic, constant thermal environment scenario. These results imply that the progressive forest expansion in abandoned agricultural lands widely recorded in European Mediterranean environments [e.g. 21] and leading to colder-dwelling communities (see Figure 2) would tend to compensate or reverse the expected responses of biological communities to global warming. For example, Gil-Tena *et al.* [22] show how large-scale forest expansion and maturation in Catalonia has favored the expansion of many forest bird species, most of which have on average cold temperature niches and northern distribution ranges. When assessing the impacts of climate change through community indicators, it should be therefore crucial to account for land use dynamics, especially in areas experiencing net forest gain [e.g. 23], where consequences of climate disruption could be underestimated. On the other hand, the occurrence of a wildfire in a forest environment, a temporally punctual event, would produce a sudden increase in the average temperature niches and a decrease in the average latitudinal ranges of bird communities. In these cases, perceived climate change impacts may be overestimated in areas strongly affected by altered fire perturbation regimes [24]. It has already been suggested that bird species and communities may respond more strongly to habitat than to climatic requirements [25]. Here we report that these responses could result in biased estimations of climate change-related impacts due to unforeseen effects of land use changes.

The basic assumption of community-based climate change indicators is that generalized warming causes non-random species distribution shifts, with warm-climate species substituting colder-climate species within local assemblages [2]. As shown in the results with our European species pool, this species turnover can be also anticipated, promoted or even reversed from changes in land use. On the other hand, biodiversity indicators based in trends of species with high habitat specialization, such as the farmland bird indicator [26], could be affected not only by land use dynamics but also by climate change, due to the complex interactions between climate and land uses [27], [28]. An interesting issue that remains to be tested is whether climate change indicators based in the integration of large-scale species trends, such as the Climatic Impact Indicator [8], are also dependent on land use dynamics. Due to its integrative formulation, using data on species trends from many countries, the index proposed by Gregory *et al*. [8] would probably be less sensitive to landscape changes than indexes based on the composition or structure of local communities. However, if land use dynamics affected large areas within a specific territory (e.g. Europe in the case of [8]) or followed different trends among different portions of that territory (e.g. Northern and Southern Europe) the sensitivity of trends-based indexes to detect impacts related to climate change could also be hindered. For example, if, as happens in Catalonia [e.g. 22], forest, cold-dwelling bird species tend to have positive trends due to forest expansion and maturation, indexes integrating species trends would tend to underestimate the impacts of climate change.

We have shown that mean climatic envelope of species can be largely dependent on land uses and disturbance regimes and that this can have important effects in our ability to detect the effects of climate change through community indicators. Our results come from an area with specific climatic, biological and socioeconomic contexts, but previous works suggest that they could be extrapolated to the whole Mediterranean Basin. Prodon [10] gave a first example on the habitat-dependant variation in the climatic niche of birds in Europe, showing that bird species occupying holm-oak (*Quercus ilex*) woodlands in France had more northerly distributions (6° on average) than those occupying adjacent grassy and stony habitats. Covas & Blondel [29] further showed that Mediterranean open-habitat bird species (including steppe, shrub and saxicolous species) tended to have more southern distribution barycentres than forest birds, which tend to be widely distributed throughout Palearctic forests. Suárez-Seoane *et al*. [11] found that bird species with a Eurosiberian distribution tended to prefer wooded areas, while Mediterranean species favored open and shrubland habitats. Moreover, it seems likely that similar interactive effects of different components of global change on climate change indicators (or on biodiversity indicators in general) could be a generalized phenomenon. In fact, the possible confounding effects of anthropogenic habitat changes on processes supposedly linked to climate change impacts have been also highlighted in other areas, e.g. the poleward shifts of the ranges of North American birds [30] or the upward elevational shift of birds in the Italian Alps [25].

Popy *et al.* [25] claimed that predictions of climate change impacts based on the climate envelopes of species should be treated with caution until the mechanisms underlying the observed patterns are better understood. Our results highlight the need to explicitly account for the interactive nature of different global change processes in order to obtain ecologically meaningful indicators of their effects on communities. This could be achieved by the integration of land use dynamics in the interpretation of the temporal variation of climate change indicators at the local community level. In this sense it would be useful to analyze the long-term trends of community climate-related indicators in areas with known trajectories of habitat characteristics. The most stable areas, those where land uses follow minimal or no changes, would probably give the best possible account of climate change impacts, offering a baseline to analyze the variation of climate change indicators in more dynamic areas. We suggest that the relative effects of climate and land use changes in a given area would be best described by reporting the variation of climate change indicators together with explicit assessments of the magnitude of land use and climatic changes. More work is needed to understand what current climate change indicators are indicating and to isolate real climate change impacts.

## Supporting Information

Figure S1 **Relationships between altitude and landscape gradients.** Relationships between altitude (up to 2000 m) and the three landscape gradients analyzed in this study. Vertical dotted lines indicate the upper altitude limit used in each case to avoid confounding effects of gradients and altitude on dependent variables of interest, due accumulation of forest of forested 1×1 km grid cells at high altitudes.
(DOC)

Table S1 **Regression coefficients describing the variation of climate change indicators along environmental and temporal gradients in Catalonia and France.** Equations from Catalonia derive from linear relationships observed using a dataset of 2824 1×1 km cells up to 2000 m above sea level. Regression coefficients corresponding to French data are those calculated by Devictor et al. [9] for presence-absence data (and thus comparable to those in the Catalonian dataset). Intercepts, coefficients of determination and associated *P*-values are also given whenever available.
(DOC)
