# Supplementary material for: Climate Change or Land Use Dynamics: Do We Know What Climate Change Indicators Indicate?
Source: PLoS One. 2011 Apr 21;6(4):e18581. doi: 10.1371/journal.pone.0018581 (PMC3080866; doi:10.1371/journal.pone.0018581)
Supplement: Figure S1 — Relationships between altitude and landscape gradients. Relationships between altitude (up to 2000 m) and the three landscape gradients analyzed in this study. Vertical dotted lines indicate the upper altitude limit used in each case to avoid confounding effects of gradients and altitude on dependent variables of interest, due accumulation of forest of forested 1×1 km grid cells at high altitudes. (DOC) [file pone.0018581.s001.doc]

**Figure S1**. **Relationships between altitude and landscape gradients**

Relationships between altitude (up to 2000 m) and the three landscape gradients analyzed in this study. Vertical dotted lines indicate the upper altitude limit used in each case to avoid confounding effects of gradients and altitude on dependent variables of interest, due accumulation of forest of forested 1×1 km grid cells at high altitudes.
