# Supplementary material for: Climate Change or Land Use Dynamics: Do We Know What Climate Change Indicators Indicate?
Source: PLoS One. 2011 Apr 21;6(4):e18581. doi: 10.1371/journal.pone.0018581 (PMC3080866; doi:10.1371/journal.pone.0018581)
Supplement: Table S1 — Regression coefficients describing the variation of climate change indicators along environmental and temporal gradients in Catalonia and France. Equations from Catalonia derive from linear relationships observed using a dataset of 2824 1×1 km cells up to 2000 m above sea level. Regression coefficients corresponding to French data are those calculated by Devictor et al. [9] for presence-absence data (and thus comparable to those in the Catalonian dataset). Intercepts, coefficients of determination and associated P-values are also given whenever available. (DOC) [file pone.0018581.s002.doc]

**Table S1. Regression coefficients describing the variation of climate change indicators along environmental and temporal gradients in Catalonia and France.**

Equations from Catalonia derive from linear relationships observed using a dataset of 2824 1×1 km cells up to 2000 m above sea level. Regression coefficients corresponding to French data are those calculated by Devictor et al. [9] for presence-absence data (and thus comparable to those in the Catalonian dataset). Intercepts, coefficients of determination and associated *P*-values are also given whenever available.

| **Predictor** | **Area** | **Dependent var.** | **Intercept** | ***b*** | ***R*^2^** | ***P*** |
| --- | --- | --- | --- | --- | --- | --- |
| Altitude (m) | Catalonia | CTIcat (ºC) | 16.77 | -0.00163 | 0.80 | < 0.001 |
|  |  | CTIeur (ºC) | 13.69 | -0.00069 | 0.44 | < 0.001 |
|  |  | CAL (º) | 43.95 | 0.00300 | 0.46 | < 0.001 |
| Average Temp. breeding season (ºC) | Catalonia | CTIcat (ºC) | 11.57 | 0.2705 | 0.83 | < 0.001 |
|  |  | CTIeur (ºC) | 11.37 | 0.1218 | 0.51 | < 0.001 |
|  |  | CAL (º) | 53.94 | -0.5242 | 0.53 | < 0.001 |
| Year (1989-2006) | France | CTIeur (ºC) |  | 0.0044 |  | < 0.001 |
| Latitude (100 km units) | France | CTIeur (ºC) |  | -0.102 | 0.51 | < 0.001 |
